# Balancing conservation and human access to nature: the impact of a constructed causeway on water levels and sedimentation, North Bull Island, Ireland – ERRATUM

**DOI:** 10.1017/cft.2025.10006

**Published:** 2025-05-30

**Authors:** I. Möller, K. O’Leary

**Affiliations:** Department of Geography, School of Natural Sciences, Trinity College Dublin, Dublin, Ireland

## Abstract

**Figure:**
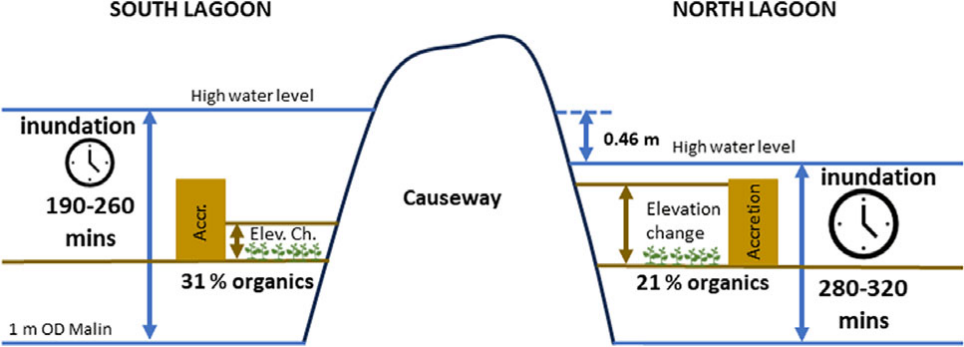

When this article was originally published in Cambridge Prisms: Coastal Futures it omitted to include the below graphical abstract.

This article also included the incorrect version of Figure 1. This can be seen on next page.

**Figure 1.**
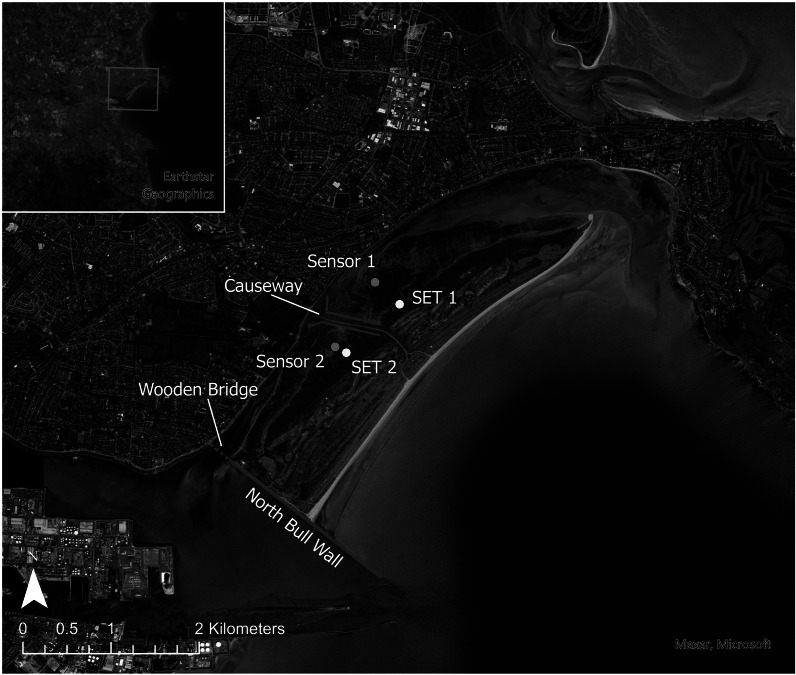
(a) location of Dublin on the east coast of Ireland (inset) and (b) the position of sedimentation elevation tables (SET) sites and water level sensors on North Bull Island, as well as the position of the Dublin Port tide gauge.

The publisher apologises for these errors.
